# Did an Intervention Programme Aimed at Strengthening the Maternal and Child Health Services in Nigeria Improve the Completeness of Routine Health Data Within the Health Management Information System?

**DOI:** 10.34172/ijhpm.2020.226

**Published:** 2020-12-05

**Authors:** Benjamin Uzochukwu, Tolib Mirzoev, Chinyere Okeke, Joseph Hicks, Enyi Etiaba, Uche Obi, Tim Ensor, Adaora Uzochukwu, Obinna Onwujekwe

**Affiliations:** ^1^Department of Community Medicine, College of Medicine, University of Nigeria (Enugu Campus), Nsukka, Nigeria.; ^2^Nuffield Centre for International Health and Development, University of Leeds, Leeds, UK.; ^3^Department of Health Administration and Management, College of Medicine, University of Nigeria (Enugu Campus), Nsukka, Nigeria.; ^4^Department of Management, University of Nigeria (Enugu Campus), Nsukka, Nigeria.

**Keywords:** Health Management Information, Data Completeness, Maternal and Child, Healthcare, Nigeria

## Abstract

**Background:** During 2012-2015, the Federal Government of Nigeria launched the Subsidy Reinvestment and Empowerment Programme, a health system strengthening (HSS) programme with a Maternal and Child Health component (Subsidy Reinvestment and Empowerment Programme [SURE-P]/MCH), which was monitored using the Health Management Information Systems (HMIS) data reporting tools. Good quality data is essential for health policy and planning decisions yet, little is known on whether and how broad health systems strengthening programmes affect quality of data. This paper explores the effects of the SURE-P/MCH on completeness of MCH data in the National HMIS.

**Methods:** This mixed-methods study was undertaken in Anambra state, southeast Nigeria. A standardized proforma was used to collect facility-level data from the facility registers on MCH services to assess the completeness of data from 2 interventions and one control clusters. The facility data was collected to cover before, during, and after the SURE-P intervention activities. Qualitative in-depth interviews were conducted with purposefully-identified health facility workers to identify their views and experiences of changes in data quality throughout the above 3 periods.

**Results:** Quantitative analysis of the facility data showed that data completeness improved substantially, starting before SURE-P and continuing during SURE-P but across all clusters (ie, including the control). Also health workers felt data completeness were improved during the SURE-P, but declined with the cessation of the programme. We also found that challenges to data completeness are dependent on many variables including a high burden on providers for data collection, many variables to be filled in the data collection tools, and lack of health worker incentives.

**Conclusion:** Quantitative analysis showed improved data completeness and health workers believed the SURE-P/MCH had contributed to the improvement. The functioning of national HMIS are inevitably linked with other health systems components. While health systems strengthening programmes have a great potential for improved overall systems performance, a more granular understanding of their implications on the specific components such as the resultant quality of HMIS data, is needed.

## Background

**Key Messages**

** Implications for policy makers**

- Comprehensive data needed to inform rational health policy management and planning decisions are needed in Nigeria but rarely generated.
- Increases in data completeness is an important element of improving data quality, which improves the chances of the resultant evidence being used for policy formulation, planning and management decisions.
- Although there was improvement in the completeness of data, problems that need to be addressed persist with the country’s health management information system for example, health workers’ lack of a good understanding of what the data is used for, lack of manpower for data collection, cumbersomeness and complexity of the forms used for data collection. There is need to address these to save the scarce resources wasted in vertical programme information systems and channel these funds to better use. This will also make it more likely that the results generated will be used for decision-making at various levels.
- The results are relevant to policy-makers, and development partners who are engaged in studying and improving National Health Management Information Systems (NHMIS) as well as programme managers and planners who are interested in improving the quality of data to inform their policy and planning decisions.

** Implications for the public**

Quality data is critical to assessing both national and global burden of disease and developing public health initiatives. Improving data quality in healthcare begins by understanding the core precepts of data quality management, the value it offers, and some of the most common challenges to avoid. A health intervention programme aimed at strengthening the maternal and child health (MCH) services in Nigeria can improve routine health data used for decision by policy-makers to ensure the health of the public.

Effective policy and management decisions require good-quality evidence.^1,2^ Data from routine Health Management Information Systems (HMIS) forms is an important and routinely collected source of such evidence.^3^ HMIS are specially designed to help health planners and managers in the management and planning of health services and programmes as well as decision-making to improve the availability and quality of care.^4,5^ HMIS are also important for health system strengthening (HSS) yet, assuring the quality of health information systems remains a challenge.

 In many low- and middle-income countries, comprehensive data needed to inform rational and effective health policy management and planning decisions are not generated, but are not of sufficient quality to use for decision-making especially at the primary health center (PHC) level.^6-8^ Health data produced in low-resource settings are rarely routinely available for every population and quality issues limit their use for policy directions.^8^ As a result, many programmes particularly those financially supported by donor funding largely ignore HMIS and spend substantial resources on establishing and maintaining vertical programme information systems.

 In Nigeria, the National HMIS (NHMIS) is designed to capture 233 variables in one proforma and routinely collected. It comprises information flow from health facilities to the local government area, and then the State and Federal level. Thus data from health facilities are collated and aggregated at these different levels.^9,10^ The goal of the Nigeria HMIS was to have an effective HMIS for informed decision-making at all levels of government.^10^

 Over the years the NHMIS have been noted to be weak specifically in terms of incomplete and inaccurate data in facility paper summaries, reliability and use in supporting the health system.^11-13^ An assessment on the data quality of the routine health management information in one of the Nigerian states found poor data quality at health facility and district levels to consist of missing values, inconsistent data and poor usability.^14^

 Efforts have been made to improve the availability of high-quality data to support decision-making at all levels of the health system in Nigeria including support to Federal Ministry of Health to develop a master facility list to improve data quality which will ultimately lead to better coordination of health services.^15^ Also, the Government has made considerable investment in strengthening health information systems, including District Health Information System 2, to support performance management and service delivery to reduce preventable deaths for mothers and newborns.^16,17^

 As Nigeria embraces the Sustainable Development Goals, maternal and child health (MCH) remains a national and international priority.^18^ MCH was the HSS component of the Subsidy Reinvestment and Empowerment Programme (SURE-P) that aimed to plough back the subsidy removed from petroleum products into projects that will benefit its citizens living in rural and underserved areas.^19^ The programme started in October 2012 but the funding was suddenly withdrawn in 2015 by a newly elected government. The MCH component of SURE-P (SURE-P/MCH) involved supply and demand components. The supply component comprised infrastructural upgrade of facilities, the supply of medical and surgical consumables and increased human resources (midwives, community health extension workers, and village health workers). These health workers received training on data management including data collection, data entry and storage, analysis, and use of routine health data as part of the supply side component. This was necessary so as to track the programme inputs and health services utilization indicators. The demand component involved paying out cash to pregnant mothers to register at a health facility and complete the continuum of care (antenatal care, delivery by a skilled birth attendant, postnatal attendance for immunization and family planning). The outputs from this programme were to be captured by the health workers using the HMIS data reporting tools which were present in every PHC facility including the non-SURE-P/MCH facilities nationwide.

 Despite a compelling need for robust evidence of HMIS function, the contribution of HSS programmes (such as SURE-P/MCH) to HMIS data quality has not been sufficiently evaluated in Nigeria. Yet, ensuring the completeness of data at the source is critical to the overall quality of data available at other higher levels of the reporting system. This paper, therefore, aims to (*a*) evaluate if SURE-P improved on the completeness of MCH data at the health facilities from the HMIS (*b*) highlight issues affecting the completeness of data, and (*c*) identify a broad set of strategies which can help further improve the completeness of HMIS data and improve its potential for informing policy, planning and management decisions. The paper should be of interest and relevance to policy-makers, and development partners who are engaged in studying and improving NHMIS as well as programme managers and planners who are interested in improving the quality of data to inform their policy and planning decisions.

## Methods

###  Study Area and Setting

 This study was undertaken in Anambra State, southeast Nigeria. The state has a population of about 4.1 million and has a mix of urban and rural areas. MCH services are primarily provided from the PHCs, each of which covers a given catchment population. There are some trained (maternity homes) and untrained (traditional birth attendants, patent medicine vendors) who also offer unmonitored MCH services. In the context of the SURE-P/MCH programme, 4 PHCs are linked to a named general hospital for referral of emergency obstetric complications, and this is referred to as a cluster (4 PHCs +1 general hospital).

 Since June 2015, we evaluated the extent which and under what circumstances the SURE-P/MCH programme in Anambra state, southeast Nigeria achieved and sustained its outputs and outcomes.^20^ The project objectives and its methodological approach were reported elsewhere.^21^ The secondary analysis of MCH data from the HMIS (collected from the facility registers) is an important component of the research project. Thus, this is part of a larger study that sought to determine the effectiveness of a novel HSS and community health workers programme in improving MCH in Nigeria.

###  Study Design

 The overall study used a case study mixed-methods approach as described elsewhere.^14^ There were 12 sites studied, which comprised 3 clusters: 4 control sites, 4 SURE-P sites, and 4 SURE-P+CCT (conditional cash transfer) sites. A cluster is made up of 4 PHCs clustered around a general hospital which serves as a referral centre for emergency obstetric care.^12^ The location of these intervention clusters was entirely decided by the SURE-P project implementation unit at the federal ministry of health.

###  Data Collection

 We collected information from 2 main sources of data: quantitative and qualitative. For our quantitative data we used a HMIS-based dataset that we had previously collected for a separate study from facility registers in the PHC facilities only which covered key monthly MCH indicators. We then used this to analysed how the completeness of those indicators changed before, during, and after the SURE-P programme across the 3 cluster. This allowed us to test, in a controlled way, whether the SURE-P programme (and its termination) had any impacts on HMIS data completeness. Second, we used in-depth interviews with purposively identified key stakeholders to understand HMIS data quality in terms of completeness and its use in their policy, management and planning decisions to understand their views and experiences with the HMIS data. We did not conduct a sample size calculation given we used an existing and fixed dataset.

 In addition to the quantitative data collection, in-depth interviews were conducted with health facility workers in 2017 after the SURE-P programme. From each of the 8 PHC facilities in the 2 intervention clusters, the facility manager and a SURE-P/MCH program midwife and another health worker who may not necessarily be a SURE-P staff but from the pre-existing staff were purposively selected for the interviews giving a total of 24 health workers. These health workers were interviewed by the researchers who were trained in interviewing. Information was collected on how they collect, summarize and transmit data in the health center and what their experiences have been in doing this, including the enablers and challenges of data management.

###  Data Analysis

 To evaluate HMIS data completeness our trained researchers had previously used a standardised proforma^22^ to collect the facility-level, monthly HMIS data on 4 key MCH indicators, all measured as counts per facility (See Supplementary file 1): (1) total antenatal clinic visits (the total number of women that month who visited the PHC for any antenatal clinic meeting); (2) total postnatal clinic visits, (the total number of women that month who visited the PHC for any postnatal clinic meeting); (3) number of deliveries taken by a skill birth attendant; (4) number of pregnant women receiving 2 doses of tetanus toxoid. We collected these facility data from all 3 clusters across 3 distinct periods (dates include the entire of each start and end month listed): (1) for the 9 months before SURE-P interventions began (January 2012 - September 2012) – the “before SURE-P” period, (2) for the 32 months during which SURE-P interventions were funded and running (October 2012 - April 2015) – the “during SURE-P” period, and (3) for the 32 months after SURE-P intervention activities ended (May 2015 - December 2017) – the “after SURE-P” period. The cut-off dates used for these periods were based on expert knowledge of the programme’s running. For this study we then used these count data to create a cluster-level, monthly measure of data completeness for these indicators. We did this by calculating the monthly, cluster-level percentage of non-missing values across the 4 indicators and across the 4 PHC facilities within each cluster as:


$$\frac{n}{16}\times 100$$

where *n* = the number of non-missing values across the 4 indicators for all 4 PHC facilities per cluster. We then used a controlled, 3-period interrupted time series (ITS) analysis approach to analyse how this monthly completeness outcome varied before, during and after the SURE-P programme in (1) the SURE-P only cluster compared to the control cluster, and in (2) the SURE-P+CCT cluster compared to the control cluster, with a separate model for each comparison. We used the *itsa* function in Stata statistical software. The ITS analyses use multiple linear regression models, but with the inferential estimates (confidence intervals and *P* values) based on Newey-West standard errors to account for temporal autocorrelation (for a given lag) and heteroscedasticity. The models were tested for generalised serial correlation (using the *actest* function in Stata) and adjustments were made to the lag structure if necessary.^23^ We specified the 2 ITS models as follows:

*M*_t_ *+ β*_0_ *+ β*_1_ *T*_t_ *+ β*_2_ *Z + β*_3_ *ZT*_t_ *+ β*_4_ *X*1_t _*+ β*_5_*X1*_t_*T*_t _*+ β*_6_*ZX*1_t_* + β*_7_*ZX*1_t_*T*_t _*+ β*_8_*X*2_t _*+ β*_9_*X*2_t_*T*_t _*+ β*_10_*ZX*2_t _*+ β*_11_*ZX*2_t_*T*_t _*+ ε*_t_

 where *M*_t_ is the outcome variable measured at each month. *T*_t_ is the month since the start of the time series (1:72), *Z* is a dummy/indicator variable indicating the intervention or control cluster (control = 0, intervention = 1), *X1*_t_ is a dummy variable indicating the “during SURE-P” period (0 = before or after SURE-P period months, 1 = during SURE-P period months), and *X2*_t_ is a dummy variable indicating the “after SURE-P” period (0 = during or before SURE-P period months, 1 = after SURE-P period months). The remaining variables are interaction terms among the variables explained above.

 Therefore, the parameter estimates (*βs*) of interest for our research questions are: (1) *β*_6_, which estimates the mean change, in percentage points of completeness, for the given intervention cluster, that occurred immediately following the start of SURE-P, after controlling for the same change as estimated for the control cluster; (2) *β*_7_, which estimates how the slope/trend (linear change in percentage points of completeness per month) changed, for the given intervention cluster, between the before SURE-P period to the during SURE-P period, after controlling for the same change as estimated for the control cluster; (3) *β*_10_, which estimates the mean change, in percentage points of completeness, for the given intervention cluster, that occurred immediately following the termination of SURE-P, after controlling for the same change as estimated for the control cluster; and (4) *β*_11_, which estimates how the slope/trend (the expected linear change in percentage points of completeness per month) changed, for the given intervention cluster, between the during SURE-P period to the after SURE-P period, after controlling for the same change as estimated for the control cluster. The remaining *βs* are structural parts of the ITS models and not of direct interest for our research questions. Therefore, for simplicity we only present the results of these 4 key parameters estimates in the results tables.

 In addition to these 2 controlled ITS analyses comparing before-to-during and during-to-after SURE-P, we also did 3 uncontrolled “two-period” ITS analyses to model how the same completeness outcome changed for each of the 2 intervention clusters and the control cluster alone, and just between 2 periods: from the start of the data until the end of SURE-P (January 2012 - March 2015), and from the end of SURE-P until the end of the data (April 2015 - December 2017). These analyses allowed us to evaluate whether there were any long-term changes in key MCH HMIS indicator completeness that started before the SURE-P programme began, and if they were present in the control cluster as well as the intervention clusters. Therefore, these analyses allowed us to evaluate whether the data were consistent with any observed improvements in key MCH HMIS indicator completeness being due to secular/background changes rather than the effects of SURE-P. For simplicity we do not detail the model here. From these models the key parameter estimates we report indicate: (1) the estimated slope/trend (linear change in percentage points of completeness per month) for the before and during SURE-P period combined; (2) the estimated mean change, in percentage points of completeness, for the given cluster that occurred following the termination of SURE-P; and (3) the estimated change in slope/trend (linear change in percentage points of completeness per month), for the given cluster that occurred between the before and during SURE-P period combined compared to the after SURE-P period.

 All the in-depth interviews (n = 24) were audio-recorded and transcribed after informed consent were collected from the respondents. Interviews were transcribed and analysed manually identifying emerging themes.

## Results

 We first present the results of the quantitative analysis of how the completeness of 4 key MCH HMIS indicators varied before, during and after SURE-P in each intervention cluster compared to the control clusters, and how the completeness varied from before and during SURE-P compared to after SURE-P in each cluster alone. We then present the results exploring health workers’ perspectives on the completeness of data as a proxy for data quality, the challenges to maintaining complete data and recommendations for reducing missing and incomplete data.

 The results from the ITS analysis suggest that there is no statistically clear evidence of any additional changes in the percentage of data completeness for the key MCH HMIS indicators, either immediately after the introduction of SURE-P or immediately after the termination of SURE-P, in either the SURE-P only or SURE-P+CCT cluster, when compared to the same immediate changes observed in the control cluster (Table 1). Similarly, the results also show no statistically clear evidence of any additional changes in the trend with which key MCH HMIS indicator completeness changed over time, either after the introduction of SURE-P or after its termination, in either the SURE-P only or SURE-P+CCT cluster, when compared to the same trends observed in the control cluster.

**Table 1:** Controlled ITS Analysis of How the Monthly Percentage of PHC HMIS Data Completeness of 4 Key MCH Indicators Varied Before, During and After the SURE-P Programme in the SURE-P Only Cluster Compared to the Control Cluster and in the SURE-P+CCT Cluster Compared to the Control Cluster

**Cluster Comparison**	**Period Comparison and Type of Change**	**Coefficient (95% CI)**	***P*** ** Value**
SURE-P only vs control	During vs before: level^a^	5.22 (-8.23, 18.67)	.444
	During vs before: trend^b^	0.07 (-1.26, 1.40)	.919
	After vs during: level^a^	1.24 (-5.88, 8.35)	.731
	After vs during: trend^b^	-0.01 (-0.45, 0.43)	.970
SURE-P+CCT vs control	During vs before: level^a^	4.28 (-6.40, 14.97)	.444
	During vs before: trend^b^	-2.64 (-4.34, -0.93)	.919
	After vs during: level^a^	6.04 (0.65, 11.43)	.731
	After vs during: trend^b^	0.84 (0.46, 1.22)	.970

Abbreviations: ITS, interrupted time series; PHC, primary health centre; HMIS, Health Management Information Systems; MCH, maternal and child health; SURE-P, Subsidy Reinvestment and Empowerment Programme; CCT, conditional cash transfer. The ITS analysis is based on a multiple linear regression (OLS) model with Newey-West standard errors to correct for temporal autocorrelation and heteroscedasticity. The cluster-level outcome is the monthly percentage of complete (non-missing) values recorded for 4 key HMIS indicators (total antenatal clinic attendance, delivery with a skilled birth attendant, 2 doses of tetanus toxoid and fully immunized children) across the 4 PHC facilities within each cluster. ^a^Changes in level represent the immediate model-estimated mean change in percentage points of completeness between the end of the stated earlier time period and the start of the stated later time period for the given intervention cluster, after controlling for the same estimated change in the control cluster (ie, the difference in differences). ^b^Changes in trend represent the model-estimated mean change in the trend of the outcome (where trend = the model-predicted mean percentage point change expected in the outcome per month) between the stated earlier time period and the stated later time period for the given intervention cluster, after controlling for the same change in trend estimated for the control cluster (ie, the difference in differences).

 However, when looking at changes in the level and trend of the completeness outcome across both the period before SURE-P and the period during SURE-P combined compared to the period after SURE-P there is: (1) a statistically clear and strongly positive trend in the percentage of the completeness outcome for all 3 clusters within the period combining before and during SURE-P (Table 2), and (2) a statistically clear “levelling out” in the percentage of the completeness outcome during the period after SURE-P in all 3 clusters, due to the outcome reaching its ceiling or near its ceiling (100% completeness).

**Table 2:** Uncontrolled ITS Analysis of How the Monthly Percentage of PHC HMIS Data Completeness of 4 Key MCH Indicators Varied From Before and During Compared to after the SURE-P Programme in the SURE-P Only, SURE-P+CCT and Control Clusters Separately

**Cluster**	**Period Comparison and Change Measure**	**Coefficient (95% CI)**	***P*** ** Value**
SURE-P only	Before and during combined: trend^a^	0.96 (0.75, 1.17)	<.001
	After vs before and during: level change^b^	-4.60 (-1.18, -0.76)	.048
	After vs before and during: trend change^a^	-0.97 (-1.18, -0.76)	<.001
SURE-P+CCT	Before and during combined: trend^a^	0.49 (0.25, 0.72)	<.001
	After vs before and during: level change^b^	-4.35 (-8.63, -0.07)	.047
	After vs before and during: trend change^a^	-0.49 (-0.72, -0.25)	<.001
Control model	Before and during combined: trend^a^	0.87 (0.73, 1.00)	<.001
	After vs before and during: level change^b^	-4.34 (-8.61, -0.08)	.046
	After vs before and during: trend change^a^	-0.84 (-0.98, -0.69)	<.001

Abbreviations: ITS, interrupted time series; PHC, primary health centre; HMIS, Health Management Information Systems; MCH, maternal and child health; SURE-P, Subsidy Reinvestment and Empowerment Programme; CCT, conditional cash transfer. The ITS analysis is based on a multiple linear regression (OLS) model with Newey-West standard errors to correct for temporal autocorrelation and heteroscedasticity. The cluster-level outcome is the monthly percentage of complete (non-missing) values recorded for 4 key HMIS indicators (total antenatal clinic attendance, delivery with a skilled birth attendant, 2 doses of tetanus toxoid and fully immunized children) across the 4 PHC facilities within each cluster. ^a^Trend = the model-predicted mean percentage point change expected in the outcome per month. Changes in trend represent the model-estimated mean change in the trend of the outcome between the stated earlier time period and the stated later time period for the given cluster. ^b^Changes in level represent the immediate model-estimated mean change in percentage points of completeness between the end of the stated earlier time period and the start of the stated later time period for the given cluster.

**Figure 1:**
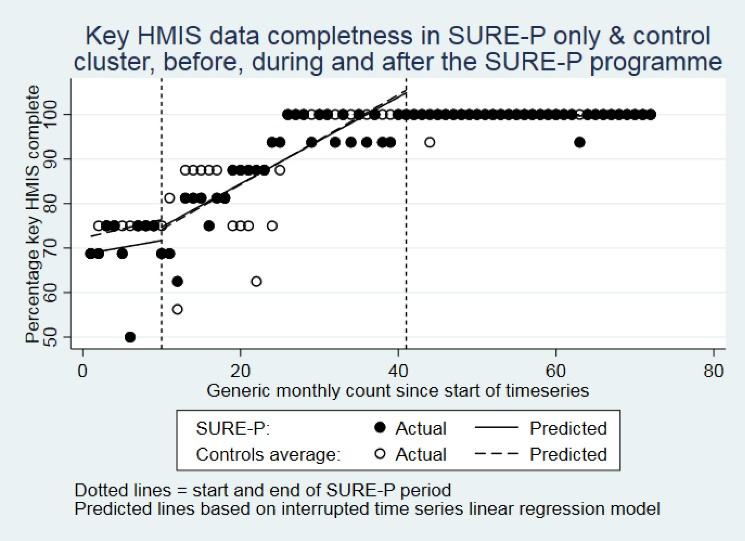
Scatter Plot of Monthly Observed Percentage Completeness for Key MCH HMIS Indicators for SURE-P Only and Control Clusters and Predicted Values From the Controlled 3-Period ITS Model. Abbreviations: ITS, interrupted time series; HMIS, Health Management Information Systems; MCH, maternal and child health; SURE-P, Subsidy Reinvestment and Empowerment Programme.

**Figure 2:**
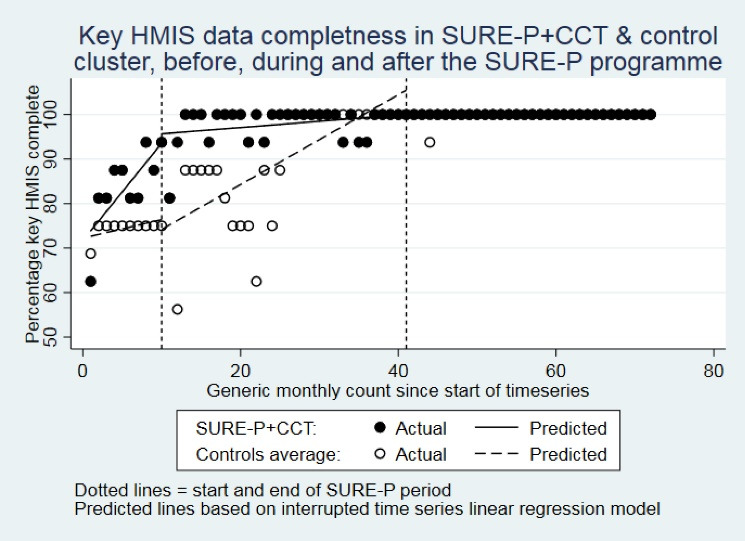
Scatter Plot of Monthly Observed Percentage Completeness for Key MCH HMIS Indicators for SURE-P Centers Giving CCT and Control Clusters and Predicted Values From the Controlled 3-Period ITS Model. Abbreviations: ITS, interrupted time series; HMIS, Health Management Information Systems; MCH, maternal and child health; SURE-P, Subsidy Reinvestment and Empowerment Programme; CCT, conditional cash transfer.

 This is also clearly apparent from Figures 1 and 2. Therefore, the results provide no evidence for any effect of SURE-P on key MCH HMIS indicator completeness within these intervention clusters, but clear evidence of a substantial positive trend in key MCH HMIS indicator completeness in all clusters that started from at least 9 months before the SURE-P period and continued for most, if not all, of the SURE-P period, and ultimately resulted in completeness improving by approximately 20%-30% points to reach typically 100% during after SURE-P period in all 3 clusters.

 At the general hospitals which were part of the intervention clusters, it was found that the NHMIS forms were not used for reporting their data, both before during and after SURE-P programme. The data from different departments of each hospital was captured in notebooks and registers supplied by other programmes, eg, the malaria and HIV control programmes. In addition to poorly filled and missing registers, there was no formal data summary or harmonization. In addition, there were discrepancies in figures between the daily records and the monthly summary records, with monthly records having comparatively larger than expected numbers for certain variables. Consequently, as many of the HMIS indicators collected in the PHC facilities were not available in the general hospitals, the general hospital data did not form part of the ITS analyses of HMIS data completeness, but clearly this is an important qualitative finding regarding HMIS data completeness in Nigerian general hospitals.

###  Health Workers’ Perspective on Completeness of Data

 During qualitative interviews, health workers felt that data completeness improved during SURE-P period and this was said to have resulted from the training given to them during the programme intervention period as well as the increased availability of staff. The participants also reflected that the quality in terms of completeness declined with the cessation of the programme. This was captured by some of the respondents thus:

*“Yes, we received training on data collection, health education, delivery and many things and this has helped us in knowing how to manage our data” *(P2C303).

*“The presence of more staffs ensured that there are more people to handle the data collection …. and the staffs were trained in data management. But after SURE-P, the data collection started declining again because we still went back to the lack of staff… we are lacking staff to handle the data” (*P2C304).

 In general, interviews showed that existing records were poorly stored after SURE-P, and staff could not account for missing registers in some of the facilities due to alleged inadequate and improper handover by exiting staff. Interviews with the facility staff revealed that data management at the PHCs entails that each staff on duty or in charge of a particular unit collects data or records activities while on duty. This is collated by the officer in charge at the end of the month, and transmitted to the state, from where it is forwarded to the federal government. This was captured by respondents thus:

*“In this facility we have staff that work in different areas in terms of data collection for antenatal clinic, Immunization, family planning etc. This is entered into different registers on a daily basis by the staff on shift and summarized at the end of the month. It is then forwarded to the local government at the end of the month by the facility in-charge” *(P2C312).

 Health workers were of the opinion that the completeness of HMIS data during the SURE-P period was as a result of more people available to handle the records and the training and re-training of staffs on data management. Other enablers of data management according to the respondents included availability of data management tools; staff motivation, which respondents pointed out that it keeps the staffs committed to the data management process; having a designated staff that is responsible for data management, mostly for the function of data collation and data summary. The enablers of data management were captured by a respondent thus:

*“Some of the things that make it easier for us are availability of registers and staff because when you don’t have adequate staff to handle it, it will be delayed …another one is the motivation of staff by making the local government accessible to us through easy transportation when returning the data” *(P2C301).

###  Challenges to Maintaining Complete Data

 The respondents stated some of the challenges they face following the halt of the SURE-P/MCH programme, noting these challenges as the reason for the discrepancies in data management. These include lack of training and retraining of staffs which make the process tedious and compromises quality; the frequency of data summary which poses a challenge due to lack of adequate human resources brought about by halting of the SURE-P/MCH programme; the fact that there is no staff dedicated to data management, which is also due to the lack of human resources; the number of variables collected in the HMIS, as the form captures 233 variables. Also, the health workers are busy with other activities in the health center, thus not having enough time for data management because of increased workload. This was noted by several respondents thus:

*“Imagine what happens when only one person is on duty and has to attend to all the patients and pregnant mothers, and still have to record data. They will record poorly and make mistakes” *(P2C307).

*“Generation of good and quality data is not easy; it is time-consuming. Sometimes the staffs don’t have enough time to see patients and they still have to document at the same time” *(P2C308).

*“There are too many things to collect information on. In our forms, we have 233 things to record and you do this on a daily and weekly basis”* (P2C302).

 Respondents were asked about the use for which data was being collected. According to most of the respondents, the information collected is used to make sure good services are delivered through informing health facility plans and staff performance management.

*“The health facility committee at times use the information to plan how to deliver good services to the people like knowing how many deliveries in a month” *(P2C317).

 However, a few respondents were unable to state the uses of the data and distinguish which data is needed for service delivery and in fact felt it was just to monitor how well they are performing in the facilities and for their promotion.

*“They just want to use the information to check how well we are performing and for our promotions” *(P2C315).

*“I don’t know which decisions they use the information for all I know is to collect the information” *(P2C312).

###  Suggestions for Improving Missing and Incomplete Data

 Some of the respondents made suggestions on how the improvement in availability and completeness of data can be solved and these included making sure that data is entered in the register from patients’ folders or files as patients arrive on a daily basis and then aggregated at the end of the month instead of waiting till the end of the month to enter the daily records from their folders to the register and then aggregate at the same time. As captured by a respondent:

*“Just as I said the data should be filled daily, do not allow it to accumulate. Accumulation of data gives problem especially in a facility that a lot of patients come in … you can do that in a facility that does not have much client, but you don’t do it in a facility that have much client load. Sometimes, you might even start looking for their missing folders and files” *(P2C305).

 Training and retraining on data management and engaging more staff were also suggested by the respondents as means of improving the availability and completeness of data. According to a respondent*: “Some of the staffs working now are untrained and needs fresh training to function well … a step-down training for the lay or volunteer staffs will help to manage the situation better” *(P2C307).

## Discussion

 We explored if the SURE P/MCH programme contributed to improving the completeness of relevant HMIS data as a proxy for improved data quality. The facility data shows that there is evidence that key MCH HMIS indicator completeness improved substantially, but these changes cannot be attributed to SURE-P alone. Therefore, there appears to have been some background changes (possibly directly and indirectly related) to SURE-P affecting HMIS data completeness. For example, there were some interventions implemented in the State by some other programmes and donors to strengthen quality improvement in PHC facilities. One such intervention was improvement of quality of care in Nigeria’s PHC facilities in rural communities between October 2013 and March 2015.^24^ The Malaria Consortium also implemented a capacity building project to improve the capacity of the national malaria elimination programme for evidence generation and use between 2008 and 2016.^25^ In addition, there were extensive trainings of health staff on monitoring and evaluation which included data management in 2010, well before commencement of SURE-P.^26-28^

 Interestingly, although data completeness improved across all the clusters, there were differences in the SURE-P and the SURE-P+CCT clusters. It is not very clear what could have contributed to the seemingly greater improvement in the CCT cluster during the intervention period. However, it is important to note that data on monthly service uptake throughout the continuum of care was collected in these CCT during quarterly monitoring and supervisory visits as they needed to track beneficiary retention throughout the period as closely as possible, and avert loss to follow-up.^29^ The supervisors also delivered spot training on record keeping during such visits. These quarterly monitoring and supervisory activities therefore may have resulted in greater improvement in data completeness in the CCT cluster during the intervention period. The non-use of NHMIS forms at the general hospitals and the preference for the use of notebooks and registers supplied by other vertical programmes is likely to lead to non-capture of the data in the NHMIS. The implication is that the data is cut off from the NHMIS and therefore cannot be used to inform planning and decision-making both at the state and country-level as the hospitals will be using different datasets. Evidence has shown that HMIS forms were less likely to be available in hospitals in Anambra State.^30^

 From health workers’ perspective, data completeness seems to have resulted from the training given to health workers during the SURE-P/MCH period as well as the increased availability of staff which was one of the intervention packages. Inadequate human resources as a result of halting of the SURE-P/MCH programme was also noted by the respondents as one of the factors affecting data completeness since there was no more staff dedicated to data management. This fact was also observed in a study where time constraints on recording tasks and the balance between recording tasks and clinic work was noted as a determinant of data completeness.^31^ However, there was no evidence to support this finding in the quantitative data which arguably provides a more accurate and objective measure of the data completeness.^32^ The in-depth staff perceptions are subjective and can be influenced by multiple biases such as their actual knowledge and expectations about data completeness and quality, and their experiences with the implementation of the SURE-P.

 From a wider perspective about the data informing decision-making, we acknowledge the emphasis in the current literature that perceptions of robust evidence by key decision-makers form important determinants of whether data (or evidence) is used to inform policy, planning and management decisions.^2,33^ Further to data completeness, such perceptions often include the source of evidence (ie, whether it comes from a reputable source such as rigorous study or established system), its comprehensiveness (ie, nationally-representative datasets often seen as better quality) and nature (‘hard’ quantitative data is often seen as better quality than ‘softer’ expert views and opinions) as well as local contents.^1,34^

 It is unclear from our study, the extent to which the design of the form to capture 233 variables in a single form contributes to the incompleteness of data. It is plausible that this burden could tire and demotivate health workers so that forms are not filled properly. Data completeness may also be reduced because most of the health workers filling the forms lack a good understanding of what the data is used for. In some settings, health workers lack an understanding of the use for which the data was being collected and are unable to distinguish which data is needed for service delivery.^35^ Similarly, it has been reported in Nigeria how the cumbersomeness and complexity of the forms, brought about by the huge number of variables was a major factor hindering the completeness of data in the NHMIS forms.^4^ Also, a 2014 review of select low- and middle-income countries by the World Health Organization (WHO) found cumulative reporting requests requiring upwards of 600 indicators^36^ and technical inconsistencies related to procedures and terms also lead to further fragmentation.^37^

 The presence of funds and human resources by SURE-P/MCH may have also contributed to a better result with the filling of the forms during the SURE-P/MCH period. However, all these were said to have decreased with cessation of the SURE-P/MCH. As noted in some African countries, human resources are a huge challenge for maintaining the quality of data within their health information systems.^38,39^ And the effect of training of health workers and increasing staff and resources for data collection has been noted as a means of improving the quality of data collected from PHCs.^40^

 Although data completeness was said to have improved we identified from health workers’ perspective that availability and completeness of data are a challenge to them, and are dependent on many health systems variables including limited staff, a high burden on providers for data collection, many variables in the data collection tools, and health worker incentives. This raises a broader point about HMIS being also linked with other health systems components and appropriate staff support and development processes and mechanisms that can be important strategies to improve HMIS data completeness. It has been noted elsewhere that factors such as lack of training, appropriate data collection tools, overwhelming task of data collection are key challenges to data quality.^41^

 Training and retraining on data management were suggested by the respondents as a means of improving the completeness of data. Evidence has shown that health HMIS training achieved an improvement in the data management practice of PHC workers.^42,43^ Integrating capacity building in HMIS strengthening efforts is an essential component of a package of HMIS strengthening interventions and are also necessary for sustainability.^44^ This has also been found to have significantly increased the completeness of the data used to monitor “prevention of mother-to-child transmission” services in South Africa.^45^

 Another strategy to improve data completeness especially at PHC level, will be to develop standard operating procedures for completing and accurately documenting in registers and monthly reporting forms. Progress towards establishing a strong, functional data collection and reporting system at PHC levels in Nigeria has been reported in a recent study that found improved data reporting and quality from the implementation of integrated community case management programs.^46^ The use of electronic or mobile devices have also been reported to reduce the burden for healthcare workers, especially community health workers at the PHC level in Nigeria^46^ and strengthening electronic health information systems, and harmonizing data collection systems have been suggested.^47^

###  Study Limitations

 Although we did not conduct a formal power calculation it is likely that our relatively small sample size meant that we lacked power to detect anything other than large changes. Also, ITS uses population-level data, so we cannot make inferences about each individual. In addition, our ITS analyses were based on only 2 SURE-P MCH clusters within one State of the Federation, and our statistical results therefore lack generalizability and are not intended to be statistically representative of all States in the Federation. There was also an imbalance in the length of the period for which HMIS data were available either side of the SURE-P period, with the pre-SURE-P period having less than a year’s worth of data available. Having access to a longer timeseries before SURE-P may have helped us understand when the improvements in HMIS completeness began, and therefore what their causes were likely to be. Also, while we attempted to assess completeness of data, we only used 4 key MCH indicators and did not conduct own observations of service provision against the recording of data. This resulted in a narrower objective measures of data completeness in our study and represents an important area for future research.

## Conclusion

 Completeness of data is an important element of data quality, which in turn will improve the chances of the resultant evidence to be used to inform policy, planning and management decisions. Although data completeness improved during SURE-P, the evidence suggests that there were no differences in improvement of data completeness between the control cluster and the SURE-P cluster; and between the control cluster and the SURE-P+CCT cluster. The observed increases in data completeness may therefore be due to other factors outside/beyond SURE-P. There were clearly factors causing improvements before and during the SURE-P period and whatever the factors, they were not harmed (or not significantly harmed) by the termination of SURE-P. However, health workers’ perceptions of how complete the HMIS data were at the relevant time periods is important and there are issues with the HMIS that need to be addressed. The functioning of national HMIS are inevitably linked with other health systems components. While health systems strengthening programmes have a great potential for improved overall systems performance, a more granular understanding of their implications on the specific components such as the resultant quality of HMIS data, is needed.

## Acknowledgements

 This work was funded by the Joint DFID/ESRC/MRC/Wellcome Trust Health Systems Research Initiative (Grant Ref: MR/M01472X/1). The funders had no role in the study design, data collection and analysis, decision to publish, or writing of the manuscript. The authors would like to thank The REVAMP Consortium project group members and the respondents for their contribution to the research.

## Ethical issues

 Ethical approvals for the study was obtained from the Health Research Ethics Committee at the University of Nigeria Teaching Hospital, Enugu (ref: NHREC/05/02/2008B-FWA00002458-1RB00002323), and the School of Medicine Research Ethics Committee at the Faculty of Medicine and Health at the University of Leeds (ref: SoMREC/14/097). Written informed consents were obtained from all the participants before data collection.

## Competing interests

 Authors declare that they have no competing interests.

## Authors’ contributions

 BU, TM, and OO designed the study. Data were collected by BU, OO, EE, CO, UO, and analysed by BU, OO, EE, CO, UO, and JH. The manuscript was drafted by BU and all authors reviewed and contributed content to the paper. BU critically revised the manuscript.

## Funding

 This work was funded by the Medical Research Council, Joint DFID/ESRC/ MRC/Wellcome Trust Health Systems Research Initiative (Grant Ref: MR/ M01472X/1 and 016530/Z/14/Z).

## Supplementary files

Supplementary file 1. Determinants of Effectiveness and Sustainability of a Novel Community Health Workers Programme in Improving Mother and Child Health in Nigeria. Proforma for Quantitative Data (PHCs).
