# Supplementary material for: Did an Intervention Programme Aimed at Strengthening the Maternal and Child Health Services in Nigeria Improve the Completeness of Routine Health Data Within the Health Management Information System?
Source: Int J Health Policy Manag. 2020 Dec 5;11(7):937–46. doi: 10.34172/ijhpm.2020.226 (PMC9808167; doi:10.34172/ijhpm.2020.226)
Supplement: Supplementary file 1 — Determinants of Effectiveness and Sustainability of a Novel Community Health Workers Programme in Improving Mother and Child Health in Nigeria. Proforma for Quantitative Data (PHCs). [file ijhpm-11-937-s001.pdf]

Supplementary file 1. Determinants of effectiveness and sustainability of a novel Community Health Workers programme in improving Mother and Child Health in Nigeria. Proforma for quantitative data (PHCs)

**Year:**														
**State:**		**LGA:**						**Facility Code:**						
**Health Facility:**		**Number of Beds:**						**Cluster Name:**						
S/№	**Categories of Indicators and their description**	**Month**												**Comments**
	**Outputs**	**Jan**	**Feb**	**Mar**	**Apr**	**May**	**Jun**	**Jul**	**Aug**	**Sep**	**Oct**	**Nov**	**Dec**	
**1**	Health facility attendance - male < 5 years of age (0-28d + 29d-11m + 12-59m)													
**2**	Health facility attendance - female < 5 years of age (0-28d + 29d-11m + 12-59m)													
**3**	Health facility attendance - male > 5 years of age (5-9yrs + 10-19yrs + 20+yrs)													
**4**	Health facility attendance - female > 5 years of age (5-9yrs + 10-19yrs + 20+yrs)													
**5**	Antenatal attendance – total													
**6**	Antenatal first visit – total													
**7**	Pregnant women that attended antenatal clinics for 4th visit during month													
**8**	Postnatal attendance – total													
**9**	Postnatal clinic visits within 1 day of delivery													
**10**	Postnatal clinic visits within 3 day of delivery													
**11**	Postnatal clinic visits within ≥7 day of delivery													

Year:		
State:	LGA:	Facility Code:
Health Facility:	Number of Beds:	Cluster Name:

S/№	**Categories of Indicators and their description**	**Month**												**Comments**
	**Outputs**	**Jan**	**Feb**	**Mar**	**Apr**	**May**	**Jun**	**Jul**	**Aug**	**Sep**	**Oct**	**Nov**	**Dec**	
**12**	Deliveries monitored using a partograph													
**13**	Deliveries taken by a skilled birth attendant													
**14**	Number of pregnant women receiving 2 doses of tetanus toxoid (TT2 for pregnant women)													
	Total number of deliveries													
**15**	Live births – Male <2.5 kg													
**16**	Live births – Male ≥2.5 kg													
**17**	Live births – Female <2.5 kg													
**18**	Live births – Female ≥2.5 kg													
**19**	Still births													
**20**	Fresh still births (FSB)													
**21**	Number of children <1 Yr of age immunised with OPV 0 birth													
**22**	Number of children < 1 Yr of age immunised with BCG													
**23**	Number of children <1 Yr of age immunised with DPT-1(penta 1)													
**24**	Number of children <1 Yr of age immunised with OPV 3													

Year:														
State:		LGA:					Facility Code:							
Health Facility:		Number of Beds:					Cluster Name:							
S/№	**Categories of Indicators and their description**	**Month**												**Comments**
	**Outputs**	**Jan**	**Feb**	**Mar**	**Apr**	**May**	**Jun**	**Jul**	**Aug**	**Sep**	**Oct**	**Nov**	**Dec**	
**25**	Number of children < 1 Yr of age immunised with DPT-3 (Penta 3)													
**26**	Number of children < 1 Yr of age immunised against measles 1													
**27**	Number of children fully immunized < 1 Yr													
**28**	(Measles 2 is not routine)													
**29**	Children < 5 Yrs placed on treatment for severe acute malnutrition (OTP & SC)													
**30**	Children < 5 Yrs discharged (as recovered) from treatment for severe acute malnutrition													
**31**	Diarrhoea new cases < 5 Yrs - male													
**32**	Diarrhoea new cases < 5 Yrs - female													
**33**	Diarrhoea new cases < 5 Yrs - male - given oral rehydration preparation (low osmolar ORS)													
**34**	Diarrhoea new cases < 5 Yrs - female - given oral rehydration preparation (low osmolar ORS)													
**35**	Diarrhoea new cases < 5 Yrs - male - given ORS and zinc supplementation													
**36**	Diarrhoea new cases < 5 Yrs - female - given ORS and zinc supplementation													
**37**	Pneumonia new cases < 5 Yrs													

Year:														
State:		LGA:					Facility Code:							
Health Facility:		Number of Beds:					Cluster Name:							
S/№	**Categories of Indicators and their description**	**Month**												**Comments**
	**Outputs**	**Jan**	**Feb**	**Mar**	**Apr**	**May**	**Jun**	**Jul**	**Aug**	**Sep**	**Oct**	**Nov**	**Dec**	
**38**	Pneumonia new cases < 5 Yrs - given antibiotics (Amoxyl DT)													
**39**	Measles new cases < 5 Yrs													
**40**	Family planning – number of male and female clients counseled													
**41**	Family planning - number of female new family planning acceptors													
**42**	Females aged 15-49 yrs using modern contraception													
**43**	Women referred out for pregnancy related complications													
**44**	Women seen and referred for obstetric fistula (VVF & RVF)													
**45**	Deaths of women related to pregnancy													
**46**	Mortality – Total deaths – Male – 0-28d													
**47**	Mortality – Total deaths – Female – 0-28d													
**48**	Persons < 5 Yrs of age with clinically diagnosed malaria													
**49**	Persons < 5 Yrs of age with confirmed uncomplicated malaria													
**50**	Persons < 5 Yrs of age with severe malaria													
